# rTMS modulates precuneus-hippocampal subregion circuit in patients with subjective cognitive decline

**DOI:** 10.18632/aging.202313

**Published:** 2020-11-30

**Authors:** Jiu Chen, Nan Ma, Guanjie Hu, Amdanee Nousayhah, Chen Xue, Wenzhang Qi, Wenwen Xu, Shanshan Chen, Jiang Rao, Wan Liu, Fuquan Zhang, Xiangrong Zhang

**Affiliations:** 1 Institute of Neuropsychiatry, the Affiliated Brain Hospital of Nanjing Medical University, Nanjing 210029, Jiangsu, China; 2 Institute of Brain Functional Imaging, Nanjing Medical University, Nanjing 210029, China; 3 Department of Neurology, Xi'an Children's Hospital, Xi'an 710003, Shaanxi, China; 4 Department of Radiology, the Affiliated Brain Hospital of Nanjing Medical University, Nanjing 210029, China; 5 Department of Neurology, the Affiliated Brain Hospital of Nanjing Medical University, Nanjing 210009, China; 6 Department of Rehabilitation, the Affiliated Brain Hospital of Nanjing Medical University, Nanjing 210029, China; 7 Department of Psychiatry, the Affiliated Brain Hospital of Nanjing Medical University, Nanjing 210029, China; 8 Department of Geriatric Psychiatry, the Affiliated Brain Hospital of Nanjing Medical University, Nanjing 210029, China

**Keywords:** episodic memory, functional connectivity (FC), hippocampal-subregion (HIPsub), repetitive transcranial magnetic stimulation (rTMS), subjective cognitive decline(SCD)

## Abstract

Hippocampal subregions (HIPsub) and their network connectivities are generally aberrant in patients with subjective cognitive decline (SCD). This study aimed to investigate whether repetitive transcranial magnetic stimulation (rTMS) could ameliorate HIPsub network connectivity by modulating one node of HIPsub network in SCD. In the first cohort, the functional connectivity (FC) of three HIPsub (i.e., hippocampal emotional, cognitive, and perceptual regions: HIPe, HIPc, and HIPp) were analyzed so as to identify alterations in HIPsub connectivity associated with SCD. Afterwards, a support vector machine (SVM) approach was applied using the alterations in order to evaluate to what extent we could distinguish SCD from healthy controls (CN). In the second cohort, a 2-week rTMS course of 5-day, once-daily, was used to activate the altered HIPsub network connectivity in a sham-controlled design. SCD subjects exhibited distinct patterns alterations of HIPsub network connectivity compared to CN in the first cohort. SVM classifier indicated that the abnormalities had a high power to discriminate SCD from CN, with 92.9% area under the receiver operating characteristic curve (AUC), 86.0% accuracy, 83.8% sensitivity and 89.1% specificity. In the second cohort, changes of HIPc connectivity with the left parahippocampal gyrus and HIPp connectivity with the left middle temporal gyrus demonstrated an amelioration of episodic memory in SCD after rTMS. In addition, SCD exhibited improved episodic memory after the rTMS course. rTMS therapy could improve the posterior hippocampus connectivity by modulating the precuneus in SCD. Simultaneous correction of the breakdown in HIPc and HIPp could ameliorate episodic memory in SCD. Thus, these findings suggested that rTMS manipulation of precuneus-hippocampal circuit might prevent disease progression by improving memory as the earliest at-risk state of Alzheimer’s disease in clinical trials and in practice.

## INTRODUCTION

Subjective cognitive decline (SCD) is characterized by a self-report of persistent memory decline whilst cognitive performance remains within the normal range [1]. It is widely accepted that SCD, which gives rise to mild cognitive impairment (MCI), is potentially the initial preclinical stage of Alzheimer’s disease (AD) [2–6]. The hippocampus, a hallmark of AD [7–9], is a prominent region which is initially involved in memory decline. Over the years, a growing body of research has consistently reported hippocampal atrophy in SCD subjects [3, 10, 11]. However, little is known about the hippocampal network connectivity of SCD subjects in the networks of hippocampal subregions. Consequently, a lack of understanding with regard to the pathophysiology of SCD hampers the development of new interventions and could prevent the clinical progression of SCD to MCI/AD.

In the past few years, neuroimaging evidence has consistently indicated the presence of afunctional heterogeneity in hippocampus subregions (HIPsub) [12–14]. A recent neuroimaging meta-analytic study divulged that the left hippocampus comprised the anterior emotional region (HIPe), middle cognitive region (HIPc), and posterior perceptual region (HIPp) based on its neurofunctional topography [12]. In addition, several studies have revealed that HIPsub topography is pathologically involved in preclinical AD [11, 14, 15]. It was also recently reported that SCD exhibited structural and functional alterations in the hippocampus [3, 10, 11, 15, 16]. Converging evidence suggested that SCD might present distinct patterns of alterations in HIPsub network connectivity. Consequently, there is mounting interest in uncovering approaches for the improvement of dysfunctional HIPsub network connectivity.

Neuromodulation techniques, including repetitive transcranial magnetic stimulation (rTMS), could provide an opportunity to modulate intrinsic connectivity networks. Over recent years, rTMS was broadly applied when investigating the changes across cortical networks [17–19]. rTMS allowed the local stimulation of an accessible network node to transmit across synapses to remote interconnected nodes with high spatial specificity [18, 20, 21]. Hence, this approach could establish a causal link between the applied stimulation and the observed changes in HIPsub network connectivity.

A previous neuromodulation study established that rTMS, which targeted the parietal cortex, improved hippocampal connectivity networks, and simultaneously ameliorated associative memory performance in healthy individuals [22]. When directed at the posterior cortical-hippocampal network, rTMS improved the precision of memory recollection [23]. In a recent MCI neuromodulation study, rTMS promoted the improvement of episodic memory by targeting the precuneus [24]. The precuneus, generally known as a remote interconnected node of hippocampal intrinsic connectivity networks [22, 25], is a critical vulnerability area for the deficit in episodic memory observed in early AD [26, 27]. Based on the above-mentioned findings, it is reasonable to speculate that whilst rTMS is directed at the precuneus in the HIPsub network, it could causally modulate the altered HIPsub network connectivity in SCD subjects.

In the present study, we first proposed a strategy to empirically investigate a pathological circuit in HIPsub related to SCD using a pattern classification approach (SVM: support vector machine). After identifying the potentially dysfunctional circuit, we aimed to activate it with rTMS in order to assess the causal links in a separate cohort. We hypothesized that SCD subjects would display distinct alterations in the patterns of HIPsub network connectivity and that these aberrations in the HIPsub circuit related to episodic memory processing could be improved by rTMS directed upon the precuneus in the HIPsub network of SCD subjects

## RESULTS

### Demographic, clinical and cognitive function characteristics

Compared with CN, SCD subjects had no significant differences in age, gender, education, general cognitive function (i.e., MMSE, MoCA, MDRS scores), and multimodal cognitive function (i.e., episodic memory, information processing speed, executive function, visuospatial function) (all *p* > 0.05, 10000 bootstraps) in the presence of higher HAMD and SCD-Q (all *p* < 0.05, 10000 bootstraps). Table 1 shows the characteristics of the study population.

**Table 1:** Demographic characteristics, clinical measures, and head rotation parameters of SCD subjects and CN.

**Items**		**CN**		**SCD**
		**n=55**		**n=38**
Age (years)		62.91(5.94)		65.84(7.73)
Gender (male/female)		23/32		8/30
Education level (years)		12.51(2.51)		12.22(2.72)
MMSE		28.58(1.43)		28.32(2.63)
MoCA		25.05(2.42)		24.92(1.79)
MDRS		141.46(2.33)		140.37(3.05)
HAMD		1.82(2.26)		3.92(3.17) ^a^
SCD-Q		3.55(1.50)		6.51(0.90) ^a^
ITV		1130.24(114.65)		1083.55(109.21) ^a^
**Episodic memory tests**				
AVLT-IR	range	10~31		11~28
	raw score	19.15(4.36)		18.66(4.22)
	*Z* score	0.35(0.94)		0.25(0.91)
AVLT-5min-DR	range	0~11		3~10
	raw score	6.35(2.20)		6.26(1.90)
	*Z* score	0.34(0.93)		0.31(0.80)
AVLT-20min-DR	range	2~10		3~11
	raw score	6.30(1.94)		6.32(2.12)
	*Z* score	0.40(0.73)		0.41(0.80)
AVLT-total	range	17~51		19~48
	raw score	31.79(7.61)		31.24(7.39)
**Composite Z scores of each cognitive domain**				
Episodic memory		0.27(0.53)		0.34(0.59)
Information processing speed		0.27(0.67)		0.18(0.71)
Executive function		0.27(0.48)		0.30(0.57)
Visuospatial function		0.17(0.66)		0.26(0.50)
**Head rotation parameters**			
FD_VanDijk		0.05(0.03)		0.04(0.03)
FD_Power		0.18(0.08)		0.16(0.09)
FD_Jenkinson		0.09(0.04)		0.09(0.05)

Data are presented as the mean (standard deviation, SD). Abbreviations: CN, healthy controls; SCD, subjective cognitive decline; MMSE, Mini Mental State Examination; MoCA, Montreal Cognitive Assessment; MDRS, Mattis Dementia Rating Scale; HAMD, Hamilton Depression Scale; SCD-Q, Subjective Cognitive Decline-Questionnaire; ITV, intracranial volume;AVLT-IR, Auditory Verbal Learning Test – immediate recall; AVLT-5min-DR, Auditory Verbal Learning Test – 5-minute delayed recall; AVLT-20min-DR, Auditory Verbal Learning Test – 20-minute delayed recall; FD, framewise displacement. ^a^ Significant differences were found between CN and SCD subjects. MMSE, MoCA, and MDRS are displayed as raw scores. This study used the composite Z scores to indicate the level of each cognitive domain. Note: this study used a re-sampling method of stationary bootstrap (10,000 bootstrap samplings) to improve the statistical power.

### Network discovery of altered HIPsub related to SCD

**Figure 1:**
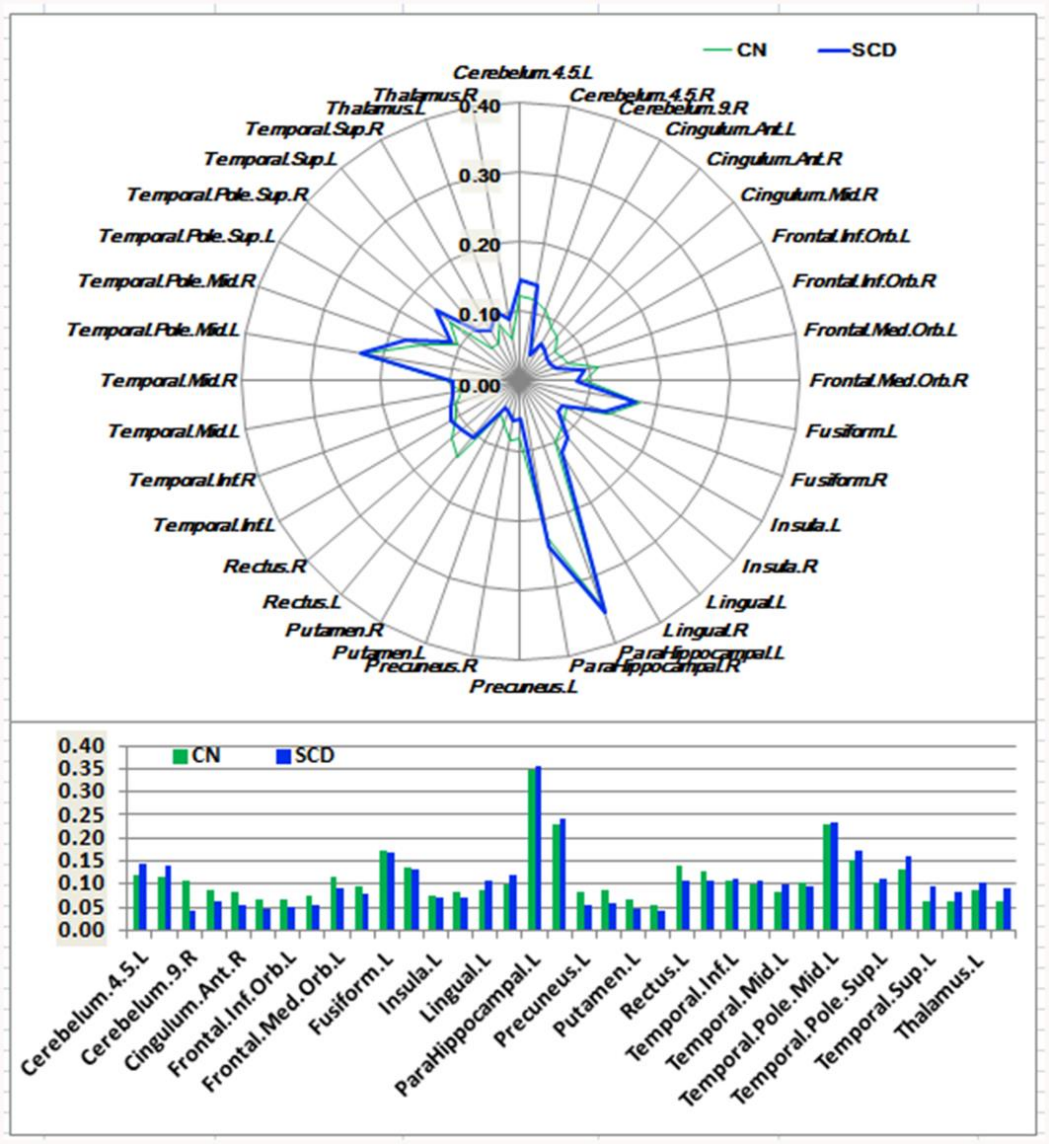
**Schematic polar plot and bar chart depicting distinct functional connectivity patterns of HIPe seeds with target regions of interest (ROI) distributed across the whole brain among CN and SCD subjects.** The concentric circles depict parameter estimates representing the connectivity strength. Note that the functional connectivity data are extracted only from the brain regions that most robustly correlated with each HIPe seed in SCD and CN, corresponding to Supplementary Figure 3. Automated anatomic labeling (AAL) atlas with 116 regions was additionally used to define the ROIs in the polar plots. Abbreviations: CN, healthy controls; SCD, subjective cognitive decline; HIPe, hippocampal emotional region; ROI, region of interest.

**Figure 2:**
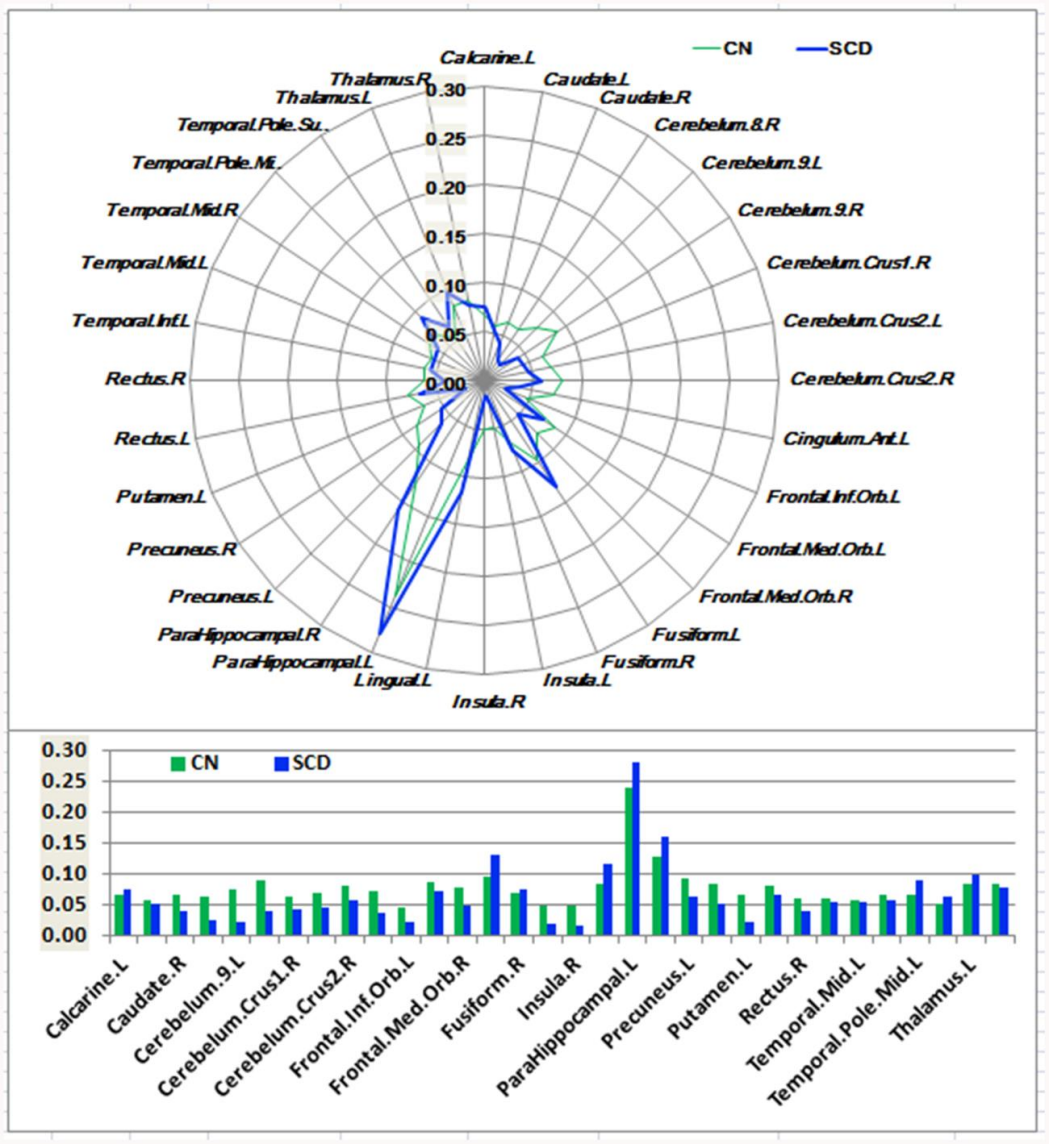
**Schematic polar plot and bar chart depicting distinct functional connectivity patterns of HIPc seeds with target ROIs distributed across the whole brain among CN and SCD subjects.** The concentric circles depict parameter estimates representing the connectivity strength. Note that the functional connectivity data are extracted only from the brain regions which most robustly correlated with each HIPc seed in SCD and CN, corresponding to Supplementary Figure 3. AAL atlas with 116 regions was also used to define the ROIs in the polar plots. Abbreviations: CN, healthy controls; SCD, subjective cognitive decline; HIPc, hippocampal cognitive region; ROI, region of interest.

**Figure 3:**
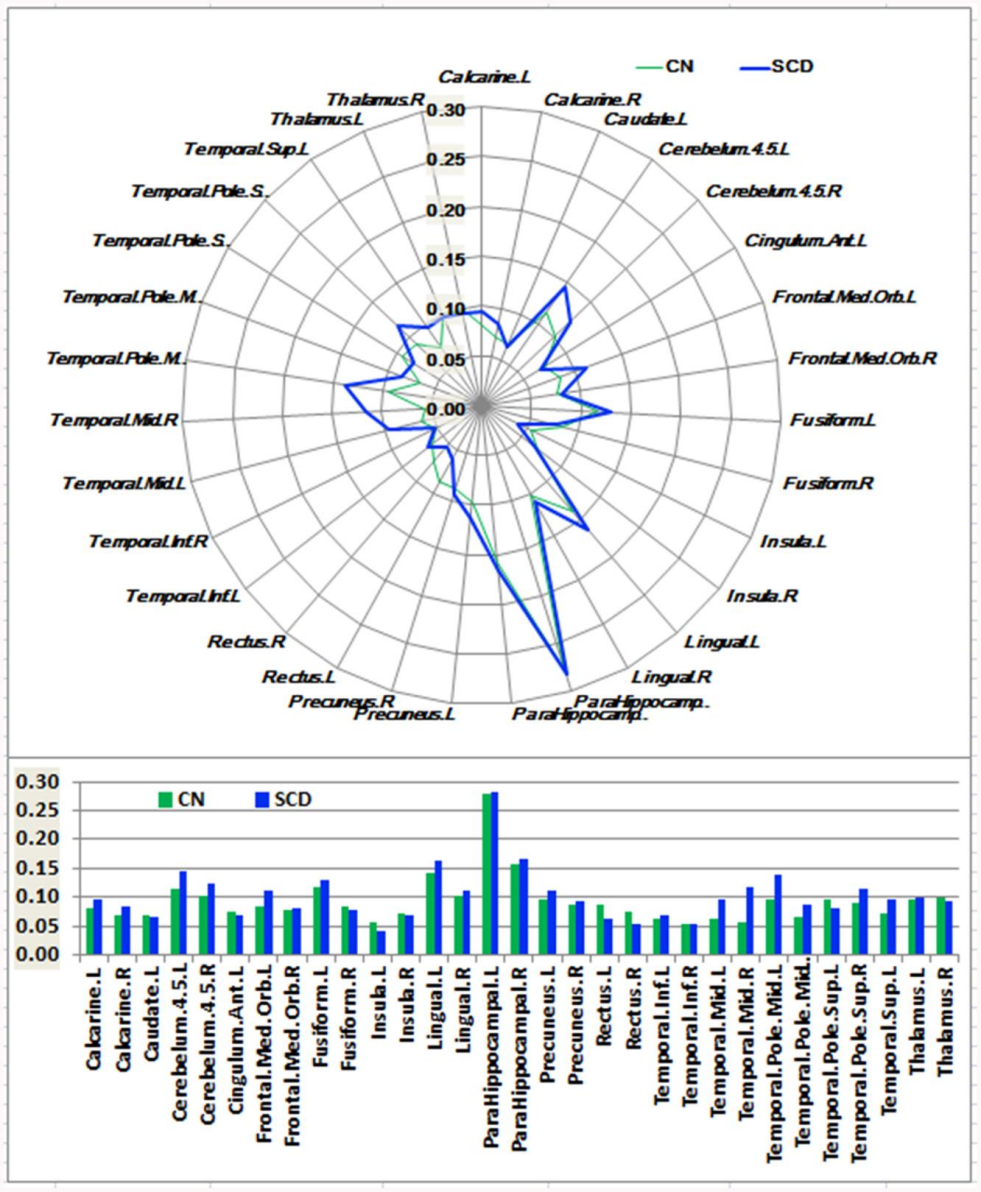
**Schematic polar plot and bar chart depicting distinct functional connectivity patterns of HIPp seeds with target ROIs distributed across the whole brain among CN and SCD subjects.** The concentric circles depict parameter estimates representing the connectivity strength. Note that the functional connectivity data are extracted only from the brain regions that most robustly correlated with each HIPp seed in SCD and CN corresponding to Supplementary Figure 3. AAL atlas with 116 regions was also used to define the ROIs in the polar plots. Abbreviations: CN, healthy controls; SCD, subjective cognitive decline; HIPp, hippocampal perceptual region; ROI, region of interest.

As shown in Figures 1–3 and Supplementary Figure 3, SCD subjects displayed distinct patterns of alterations in the HIPsub network connectivity (i.e. HIPe, HIPc, and HIPp networks) compared to CN. HIPesub region most robustly correlated with bilateral parahippocampal gyrus, left middle temporal pole, and left fusiform gyrus. HIPc subregion was more strongly correlated with bilateral parahippocampal gyrus, left lingual gyrus, left fusiform gyrus, and left thalamus. HIPp subregion most robustly correlated with bilateral parahippocampal gyrus, left lingual gyrus, and left middle cerebellum.

**Figure 4:**
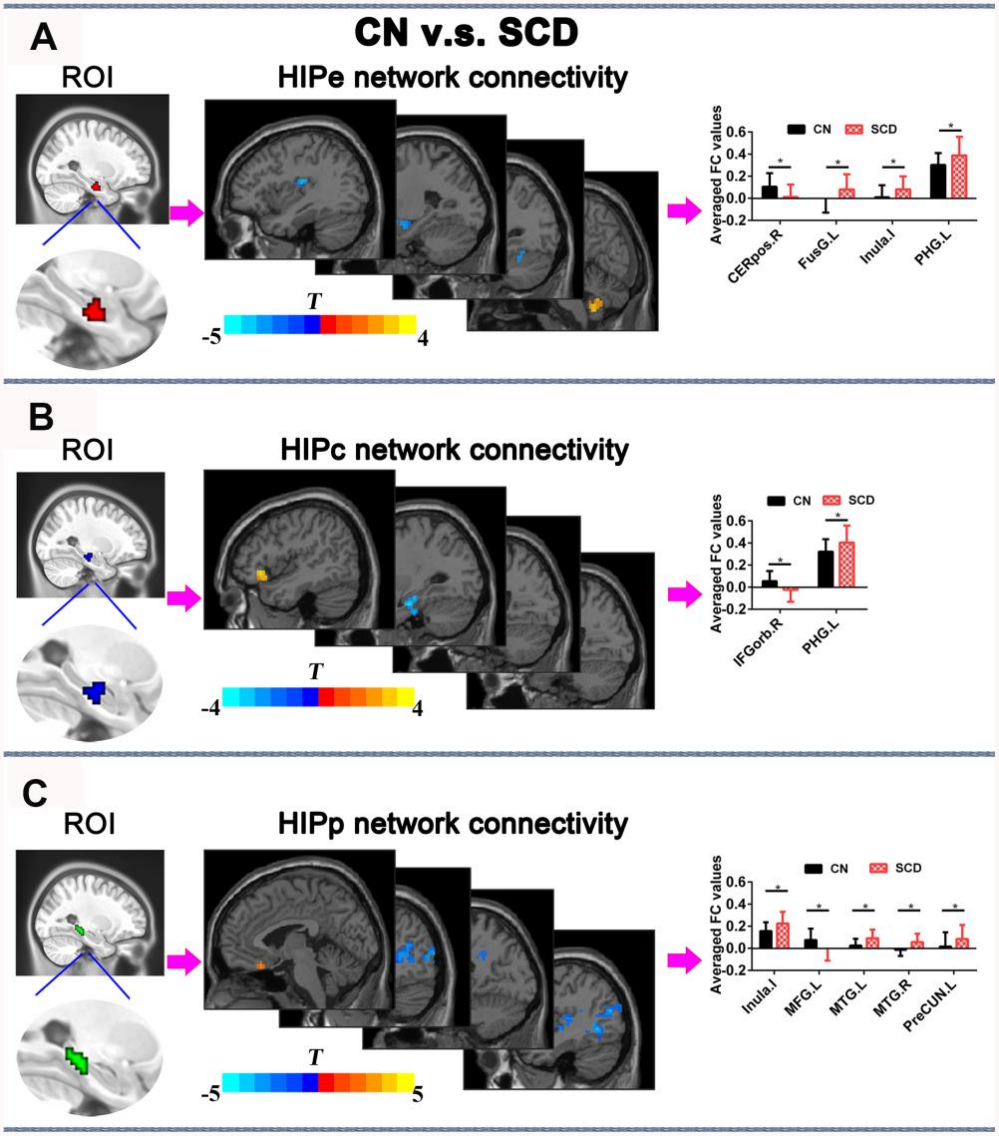
**Differences in HIPsub functional connectivity between SCD subjects and CN before rTMS treatment after controlling for age, sex, education, ITV, and FD (*p* < 0.05, TFCE-FDR correction, cluster size > 405 mm^3^.** (**A**) HIPe-subregion and different brain regions of the HIPe functional connectivity between CN and SCD subjects. The bar chart shows the quantitative comparison of functional connectivity in these regions. (**B**) HIPc-subregion and different brain regions of the HIPc functional connectivity between CN and SCD subjects. The bar chart depicts the quantitative comparison of functional connectivity in these regions. (**C**) HIPp-subregion and different brain regions of the HIPp functional connectivity between CN and SCD subjects. The bar chart indicates the quantitative comparison of functional connectivity in these regions. * *P*_TFCE-FDR_<0.05. Abbreviations: CN, healthy controls; SCD, subjective cognitive decline; HIPe, hippocampal emotional region; HIPc, hippocampal cognitive region; HIPp, hippocampal perceptual region; TFCE, threshold-free cluster enhancement; FDR, false discovery rate; ITV, Intracranial volume; FD, framewise displacement; CEREpos.R, right cerebellum posterior lobe; FusG.L, left fusiform gyrus; PHG.L, l gyrus; IFGorb.R, right inferior frontal gyrus, orbital part; MTG.R, right middle temporal gyrus; MTG.L, left middle temporal gyrus; MFG.L, left medial frontal gyrus; PreCUN.L, left precuneus.

In the HIPe network, SCD subjects exhibited reduced FC in the right cerebellum posterior lobe, and increased FC in the left fusiform gyrus, left insula, and left parahippocampal gyrus in contrast to CN (*P*_TFCE-FDR_ < 0.05, cluster size > 405 mm^3^) (Figure 4A and Supplementary Table 3). In the HIPc network, SCD had lower FC in the right inferior frontal gyrus (orbital part), and higher FC in the left parahippocampal gyrus when compared to CN (*P*_TFCE-FDR_ < 0.05, cluster size > 405 mm^3^) (Figure 4B and Supplementary Table 3). In the HIPp network, SCD had decreased FC in the left medial frontal gyrus, and increased FC in the left insula, bilateral middle temporal gyrus, and left precuneus (*P*_TFCE-FDR_<0.05, cluster size > 405 mm^3^) (Figure 4C and Supplementary Table 3). All results were controlled for age, sex, education, ITV, and FD.

### Classification of SCD patients based on the altered HIPsub GM volumes and functional connectivities

**Figure 5:**
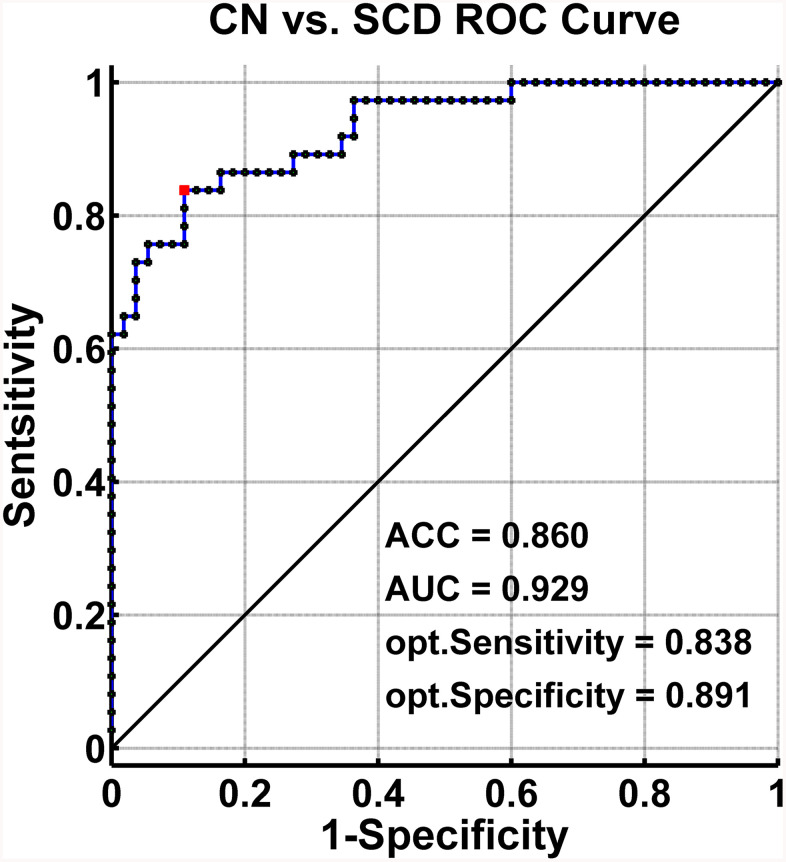
**Classification of individuals as SCD versus CN by MRI-based “classifier”.** The ROC curves hows the classification power in MRI-based “classifier” of SCD from CN. Note: the values of ACC, AUC, sensitivity, and specificity in lower right of the figure present the optimum values under the optimum combined index score (red point). Abbreviations: SCD, subjective cognitive decline; CN, healthy controls; AUC, area under the ROC curve; ACC, accuracy; Opt, optimum; ROC, receiver operating characteristic; MRI, magnetic resonance imaging.

The SVM classification had an accuracy of 86.0%. The SVM classifier’s receiver operating characteristic (ROC) curve demonstrated a high power in the discrimination of SCD patients from CN on an individual basis, with an AUC of 92.9%, 83.8% sensitivity, and 89.1% specificity as depicted in Figure 5.

### Network changes of altered HIPsub related to SCD with rTMS

### Changes in HIPsub FC pre- v.s. post-rTMS (or sham rTMS)

**Figure 6:**
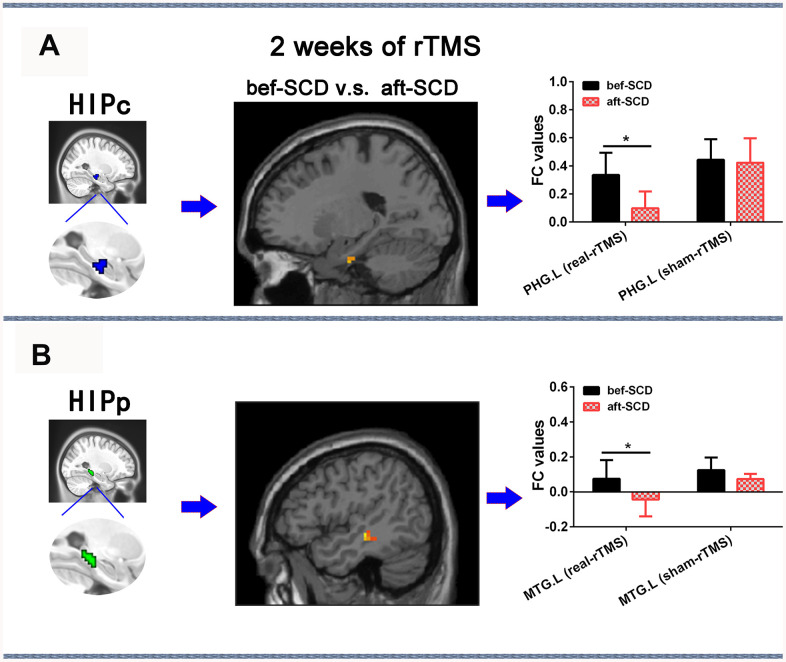
**Changes in HIPsub network functional connectivity of SCD before and after 2 weeks of rTMS treatment controlling for age, sex, GM, and education.** (**A**) HIPc seed, brain regions of HIPc functional connectivity changes, and quantitative changes on HIPc functional connectivity of SCD subjects after 2 weeks of rTMS treatment. (**B**) HIPp seed, brain regions of HIPp functional connectivity changes, and quantitative changes on HIPp functional connectivity of SCD subjects after 2 weeks of rTMS treatment. * *p*<0.05. Abbreviations: bef-SCD, subjective cognitive decline before rTMS treatment; aft-SCD, subjective cognitive decline after rTMS treatment; HIPc, hippocampal cognitive region; HIPp, hippocampal perceptual region; PHG.L, left parahippocampal gyrus; MTG.L, left middle temporal gyrus.

The 2×2 repeated-measures ANOVA showed that there were significant interactions between group (real group and sham group) and stimulation (pre-rTMS and post-rTMS) in the alterations of HIPc and HIPp network connectivities (*p* < 0.05). In the HIPc network, SCD subjects showed a significantly reduced FC in the left parahippocampal gyrus at 2 weeks of post-rTMS compared with pre-rTMS (*p*< 0.05, 10000 bootstraps) (Figure 6A). In the HIPp network, SCD subjects had significantly lower FC in the left middle temporal gyrus at 2 weeks of post-rTMS in contrast with pre-rTMS (*p*< 0.05, 10000 bootstraps) (Figure 6B). However, no differences were found in the connectivity of HIPe, HIPc, and HIPp pre- v.s. post-sham rTMS (Figure 6).

### Changes of episodic memory pre- v.s. post-rTMS (or sham rTMS)

**Figure 7:**
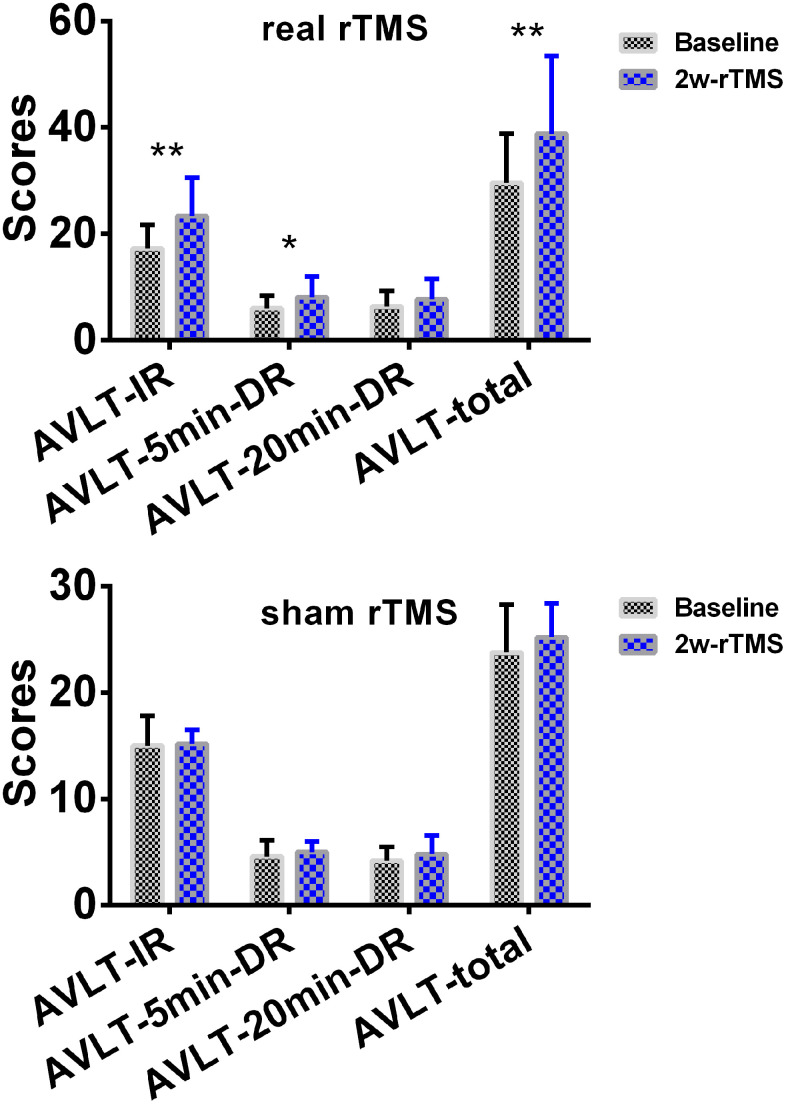
**Changes of episodic memory in SCD subjects after 2 weeks of rTMS treatment.** The line chart depicts the changes in episodic memory in SCD subjects before and after 2 weeks of rTMS treatment. To improve the statistical power, this study used a re-sampling method of stationary bootstrap (10,000 bootstrap samplings) to obtain significance between groups. * *p*<0.05, ** *p*<0.01. Abbreviations: CN, healthy controls; SCD, subjective cognitive decline; HIPc, hippocampal cognitive region; MTG.L, left middle temporal gyrus; FC, functional connectivity; 2w-rTMS, 2 weeks of rTMS. AVLT-IR, Auditory Verbal Learning Test - immediate recall; AVLT-5min-DR, Auditory Verbal Learning Test – 5-min delayed recall, AVLT-20min-DR, Auditory Verbal Learning Test –20-min delayed recall.

The 2×2 repeated-measures ANOVA also indicated that there were significant interactions between group (real group and sham group) and stimulation (pre-rTMS and post-rTMS) in the changes of AVLT-IR and AVLT-tot scores (*p* < 0.05). As shown in Figure 7, SCD subjects showed an improvement in episodic memory (AVLT) after 2 weeks ofreal rTMS treatment (10000 bootstraps; 17.25 (19.0~45.0) vs. 38.87 (16.0~59.0), T = -4.862, *p* = 0.002 for AVLT-IR; 6.00 (3.0~10.0) vs. 8.13 (3.0~12.0), T = -2.487, *p* = 0.042 for AVLT-5min-DR; 6.38 (3.0~11.0) vs. 7.38 (2.0~12.0), T = -1.283, *p* = 0.240 for AVLT-20min-DR; 29.63 (19.0~45.0) vs. 38.88 (16.0~59.0), T = -3.631, *p* = 0.008 for AVLTtot). Conversely, no differences were observed after 2 weeks of sham TMS treatment (10000 bootstraps, 15.00 (13.0~20.0) vs. 15.20 (14.0~17.0), T=-0.173, *p* = 0.871 for AVLT-IR; 4.60 (3.0~6.0) vs. 5.00 (4.0~6.0), T = -0.590, *p* = 0.587 for AVLT-5min-DR; 4.20 (3.0~6.0) vs. 4.80 (2.0~7.0), T = -0.739, *p* = 0.501 for AVLT-20min-DR; 23.80 (19.0~30.0) vs. 25.20 (21.0~29.0), T = -1.723, *p* = 0.160 for AVLTtot).

### Changes of depression scores pre- v.s. post-rTMS (or sham rTMS)

There were no significant changes in HAMD scores before and after real-rTMS or sham-rTMS (2.88 vs. 2.13, t = 1.21, *p* = 0.265 for real-rTMS group; 3.20 vs. 2.80, t = 0.590, *p* = 0.587 for sham-rTMS group) (Supplementary Figure 4).

## DISCUSSION

To the best of our knowledge, the current study was the first to demonstrate that the dysfunctions in posterior hippocampus (i.e., HIPc and HIPp) network connectivity could be causally improved byrTMS modulation upon the precuneus in SCD subjects. Our findings further suggested that the precuneus-HIPsub circuit is a potential target circuit which could prevent the clinical progression of SCD to MCI/AD in therapeutic trials.

This study demonstrated that SCD subjects exhibited distinct patterns of alterations in the HIPsub network connectivity compared to controls. The SVM classifier showed that the abnormalities in brain memory networks had a high power to discriminate SCD from CN on an individual subject basis, with an ACC of 86.0%, AUC of 92.9%, sensitivity of 83.8%, and specificity of 89.1%. The present study identified a pathological circuit in HIPsub related to SCD patients which is consistent with the notion that HIPsub is involved in functional heterogeneity [12–14].

The HIPe network connectivity in SCD subjects was predominantly abnormal in the brain regions involved in numerous aspects of emotional processing, including the cerebellum [28, 29], anterior insula [28, 30], parahippocampal gyrus [28, 31], and fusiform gyrus [32]. Since SCD subjects are characterized by memory decline [33], which significantly amplifies the risk of AD [5], the aforementioned findings support that SCD subjects commonly have an abnormal emotional processing network connectivity. SCD subjects also had altered HIPc and HIPp network connectivity in the brain regions which are involved in memory processing namely the input and integration of sensory perception spatial information, visual object recognition memory, and the formation of episodic memory [14, 34–36].

Based on the processing theory of memory system, memory formation requires two neuropathways including occipital-temporal visual object processing pathway (the “what” stream) [36, 37] and parieto-temporal visuospatial pathway (the “where” stream) [38, 39]. Therefore, our results suggested that although self-reported memory declined within the normal range, there were abnormalities in the networks associated with memory processing in SCD patients. Hence, it can be inferred that aberrations in memory processing networks might precede the onset of clinical symptoms in SCD subjects.

The most interesting finding of the current study was that rTMS modulation directed upon the precuneus for 2 weeks could improve HIPc connectivity with the left parahippocampal gyrus and HIPp connectivity with the left middle temporal gyrus, potentially resulting in an improvement of episodic memory. Indeed, numerous studies have consistently indicated that the precuneus is a key node which forms part of the intrinsic hippocampal connectivity networks [14, 22, 25]. It was also previously reported that the precuneus serves as a key node for episodic memory deficits observed in early AD [26, 27], and is vulnerable to disruption which leads to the progression of MCI to AD [24]. Recent studies described the functional organization and connectivity in the human posteromedial cortex where the precuneus is located [40]. Moreover, the anatomical connectivity patterns of the precuneus potentially reflected its functional architecture [41].

In the past, the local rTMS stimulation of accessible network nodes was not only transmitted across synapses to distant parts but also to highly spatially specific interconnected nodes [18, 20, 21]. A recent task fMRI study has demonstrated a brain-behavior relationship whereby the structural network system dynamics showed direct relationships with brain activity associated with working memory [42]. Therefore, it is reasonable to speculate that the effects of rTMS might propagate in the hippocampus through synaptic transmission in the precuneus-HIPsub pathway.

The present study showed that rTMS modulation could improve the dysfunctions in posterior hippocampus (i.e., HIPc and HIPp) network connectivity related to memory processing but could not ameliorate the dysfunctions in anterior hippocampus (i.e., HIPe) network connectivity related to emotional processing. Previous studies have also indicated that the precision of memory recollection could be improved by stimulating the posterior cortical-hippocampal network [23] and parietal cortex connectivity with the hippocampus using rTMS [43]. Therefore, the precuneus-HIPsub pathway is believed to be an ideal target circuit for tailored rTMS intervention which could improve episodic memory decline in SCD.

The current study also showed that SCD subjects had significantly higher HAMD scores compared to controls although none of the subjects were depressed (HAMD score < 7). This could be explained by the confounding effect of depression on HIPsub connectivities alterations in SCD patients. Recently, Liew and colleagues investigated the independent risks of neurocognitive disorders associated with depression in a large sample study. They revealed that SCD and depression were independent risk factors for MCI/dementia and the combination of depression with SCD posed a higher risk than SCD alone [44].

Furthermore, several studies have indicated that TMS, such as electroconvulsive therapy (ECT), could be used for the treatment of depression [45–47]. However, to avoid depression as a confounding factor, only subjects with HAMD scores of less than 7 were recruited in the present study. Additionally, no correlations were found between depression scores and HIPsub connectivities. Our study also demonstrated that there were no significant changes in depression scores before and after real-rTMS or sham-rTMS. Therefore, it is plausible that the dysfunctions in HIPsub connectivities were not caused by depression in our study. In the future, depression scores between SCD and normal subjects should be further when investigating the effects of TMS on SCD subjects.

### Limitations

There are several limitations to the present study. Firstly, a relatively small sample size was used. Based on the rTMS clinical trials, a single clinical symptom indicator was utilized as the clinical efficacy criteria. However, our results are reliable since we combined clinical response with functional connectivity as the efficacy criteria. In our study, rTMS was potentially efficient in restoring the dysfunctions in HIPc and HIPp network connectivity while HIPe network connectivity dysfunction persisted. In the future, a larger sample size is needed to explore the therapeutic pathways pertaining to the network connectivity of anterior hippocampus. Lastly, this study only investigated high-frequency effects [48]. Previous studies indicated that there were frequency-specific neuromodulation effects on improving episodic memory. Future studies need to further explore low-frequency effects on the improvement of episodic memory.

## CONCLUSIONS

This study provides a novel experimental evidence on the correction of an aberration in the posterior hippocampus (HIPc and HIPp) related to cognitive and perceptual processing by modulating the precuneus in SCD patients. rTMS manipulation might prevent disease progression by improving memory in the earliest at-risk state of AD during clinical treatment trials. Our study further suggested that the precuneus-HIPsub circuit might be a useful target circuit for SCD subjects to design rationale strategies for therapeutic trials.

## MATERIALS AND METHODS

In the first cohort, a total of 99 subjects, comprised of 42 SCD patients and 57 healthy controls (CN), participated in the current study. All participants were selected from our in-home database: the Nanjing Brain Hospital-Alzheimer’s Disease Spectrum Neuroimaging Project (NBH-ADsnp) (Nanjing, China). Relevant information pertaining toNBH-ADsnpis summarized in supplementary materials (*SI Methods S.2.*). 3 SCD patients and 2 healthy controls were discarded from further analyses due to excessive head movement (see quality assurance section below), and incomplete or missing MRI data. The final analyses included 38 SCD patients and 55 CN. The detailed inclusion and exclusion criteria are described in supplementary materials (*SI Methods S.2*).

In the second cohort, a total of 20 SCD subjects, selected from the NBH-ADsnp database, participated in the clinical trial (No. ChiCTR2000034533). The participants underwent a 5-day, once-daily rTMS (or sham) course for 2 weeks in order to stimulate the precuneus in a sham-controlled design. Clinical measures, neuropsychological assessments, and MRI data were collected at baseline (pre-rTMS or sham intervention) and at the end of the 2 weeks of sham stimulation. Among the 20 SCD participants enrolled in the study, 16 subjects were randomly divided into real rTMS (8 SCD) or sham (8 SCD), and 13 subjects (8 SCD for real rTMS, 5 SCD for sham rTMS) completed the 2-week trial of rTMS course.

### Ethical principle

This study was approved by the Human Participants Ethics Committee of the Affiliated Brain Hospital of Nanjing Medical University (No. 2018-KY010-01, No. 2020-KY010-02, and No. ChiCTR1900022287). Written informed consents were obtained from all subjects.

### Neuropsychological assessments

In the present study, a standardized clinical interview and comprehensive neuropsychological assessment were performed in order to evaluate general cognitive function (MMSE, MoCA, and MDRS), executive function (TMT-B, Stoop-C, DST, VFT, and Similarity), information processing speed (TMT-A, Stoop-A, Stoop-B, and DSST), episodic memory (AVLT, LMT, and ROCFT-20-min-DR), and visuo-spatial function (ROCFT and CDT). Comprehensive neuropsychological assessments are summarized in supplementary materials (*SI MethodsS.3*).

### MRI data acquisition

Detailed MRI data acquisition parameters included in the NBH-ADsnp database are summarized in supplementary materials (*SI MethodsS.4*).

### fMRI data preprocessing

In this study, MATLAB 2015b and DPABI software [49] were used to preprocess all fMRI data. The image processing procedures were performed as described by Yan et al. [50] and are summarized in supplementary materials (*SI Methods S.5*). Overall, the image processing procedures included slice timing correction, head motion correction, realignment, nuisance covariate regression, normalization, smoothing, and filtering.

### Quality assurance (QA)

### Brain atrophy effect

Since significant hippocampal GM atrophies in SCD subjects were reported in the past [11, 51], the anatomical differences between our groups might influence the FCs of HIPsub. In order to elucidate this matter, we computed global intracranial volumes (ITV) based on native GM, WM, and CSF in both CN and SCD subjects by using in-home MATLAB codes. Furthermore, when computing the general linear model (GLM), ITV was taken as an additional covariate in order to investigate network connectivity differences of HIPsub between CN and SCD subjects.

### Head motion effect

Three approaches were employed to control the effects of head motion, both at individual and group levels. Firstly, SCD subjects with excessive head motions (cumulative translation or rotation > 3.0 mm or 3.0°) were excluded. Afterwards, a Friston 24-parameter model was used to regress out head motion effects from the realigned data [52]. Secondly, a ‘scrubbing’ procedure was performed to scrub frames (volumes) with an excessively high whole-brain root mean square (RMS) signal change over time in the preprocessed fMRI data for each individual [53–55]. Furthermore, all volumes were regressed out with a framewise displacement (FD) greater than 0.2 mm as nuisance covariates, and any scan with 50% of volumes removed was discarded [56]. 1 CN was excluded due to excessive head movement. There were no significant differences in the head motion parameters between the subsequently included CN and SCD subjects (Table 1).

### Strict multiple comparison correction strategy

A strict multiple comparison correction was performed in order to ensure reproducibility, test–retest reliability, and replicability on fMRI metrics [57]. Statistical maps were leveled using permutation test with Threshold-Free Cluster Enhancement (TFCE) [58] and false discovery rate (FDR) was implemented in DPABI [49]. For cluster-extent permutation tests, voxel thresholds were set as two-tailed, *p*< 0.02 (*Z*> 2.3). Lastly, we set a two-tailed, *p*< 0.05 threshold for our analyses (1,000 permutations in FDR evaluation).

### Definition of hippocampal subregions

The definition of HIPsub employed throughout our study was originally designated by Robinson et al. [12] and Bai et al. [59], who previously used coactivation-based parcellation to reveal a subspecialization in the hippocampus using a data-driven method. Since both studies demonstrated that the right hippocampal segmentation was ambiguous using coactivation-based parcellation, we selected only the left HIPsub as regions of interest (ROI) (Supplementary Figure 2). The left hippocampus was then defined as three subregions (HIPe, HIPc, and HIPp).

### Functional connectivity analyses

Firstly, we extracted the average time courses for all voxels within each HIPsub as the reference time course. Secondly, we performed voxel-wise cross-correlation analysis between the averaged time courses of all voxels within the seed HIPsub region and each voxel in the remainder of the whole brain within the group-specific GM mask. At last, we performed a Fisher's z-transform analysis to enhance the normality of the correlation coefficients.

### rTMS protocol

rTMS was employed to stimulate the precuneus of all SCD participants using a Magstim Rapid2 magnetic stimulator with a 70-mm figure-8-shaped coil. The Pz site of the 10–20 electroencephalogram system was used to locate the precuneus, and the intersection tip of the two coil loops was placed at the Pz site to stimulate the precuneus [24].

rTMS was then applied at a frequency of 10 Hz, using total trains of 1000 stimuli (1000 pulses) and at an intensity of 100% of the motor threshold (MT). The MT was defined as the lowest intensity producing motor evoked potentials of greater than 50 μV in at least 5 out of 10 trials in the relaxed first dorsal interosseous (FDI) muscle of the contralateral (right) hand [60]. All participants received a total of 25 sessions of either rTMS or sham stimulation over the precuneus. Daily session consisted of a 4 s stimulation with an interval of 56 s. The entire session lasted for 25 minutes each day. The subjects received 5 sessions per week for 4 weeks (Monday-Friday for a 4-week period). In the sham rTMS group, the stimulation coil was flipped over (180 degrees from the original position) to provide an identical sound. The flipped coil also induced a tapping sensation on the scalp.

### Adverse events of rTMS protocol

The participants did not report any adverse effects during the rTMS trial.

### Statistical Analysis

### Demographics and neuropsychological data

Statistical analyses were conducted by the Statistical Package for the Social Sciences (SPSS) software version 22.0 (IBM, Armonk, New York). Two-sample t-test and chi-square tests were computed so as to assess differences in demographic data, clinical, cognitive performance, ITV, and head rotation parameters between the SCD and CN subjects (*p* < 0.05).

### Altered HIPsub network related to SCD

In order to characterize the FC patterns of HIPsub network at a group level, we performed a random-effect analysis using one-sample *t*-tests in the spatial maps of FC in CN and SCD subjects with a stringent threshold set at *p* < 0.001 using permutation test with TFCE as well as family-wise error (FWE) correction together with a cluster extent k > 100 voxels (2700 mm^3^). Afterwards, masks were created based on brain regions that most robustly correlated with each HIPsub seed in SCD and CN subjects. The functional connectivity data were extracted only from the brain regions within these masks. Schematic polar plots were used to describe FC patterns of each HIPsub seed with target regions throughout the whole brain and could characterize abnormal FC patterns of HIPsub seeds to specific target brain ROIs. Furthermore, we used the automated anatomic labeling (AAL) atlas with 116 regions in order to define the ROIs in the polar plots. All brain figures were generated using the DPABI software based on SPM8 [49]. GraphPad Prism 6.0 was applied to generate the bar graphs. The polar plots were drawn using Microsoft Excel 2007 software.

GLM analysis was performed to investigate the differences in the FCs of HIPsub between SCD and CN subjects before rTMS treatment, after controlling for age, sex, education, ITV, and mean FD (TFCE-FDR-corrected *p*< 0.05 and cluster size > 405 mm^3^). We then constructed masks based on brain regions which showed differences in the FCs of HIPsub in SCD compared to CN. These masks were used for the analysis of pre- v.s. post-rTMS (pre-sham- v.s. post-sham-rTMS) fMRI data from cohort 2 (i.e., network changes of altered HIPsub related to SCD). The results demonstrated an altered network connectivity in HIPsub related to SCD during rTMS treatment.

### Pattern classification based on altered HIPsub GM and FC

A support vector machine (SVM) approach was applied to further identify GM and network connectivity of altered HIPsub in SCD patients using the alterations in the identified ROIs as a biomarker to evaluate the extent to which we could distinguish SCD from CN subjects. A leave-one-out cross-validation (LOOCV) strategy was used to assess the generalization of this SVM classifier and measure its accuracy, sensitivity, and specificity. These findings demonstrated the presence of GM and network connectivity of altered HIPsub related to SCD patients and could explain the changes associated to SCD during rTMS treatment.

### Changes in episodic memory, depression score, and network of altered HIPsub related to SCD with rTMS

Paired t-tests were used to calculate the changes in episodic memory and HAMD scores pre- v.s. post-rTMS (or pre-sham- v.s. post-sham-rTMS) in SCD subjects in an attempt to investigate the improvement of episodic memory and depression score. Paired t-tests were also performed to analyze the changes in network FC of HIPsub pre- v.s. post-rTMS (or pre-sham- v.s. post-sham-rTMS) in SCD subjects so as to empirically investigate altered HIPsub network connectivity related to SCD, after controlling for age, sex, education, and GM.

### Sham v.s. real rTMS

Among the 13 SCD subjects with complete clinical assessments, usable sMRI and fMRI scan data at baseline, and 2 weeks of post-rTMS (or sham), 8 subjects were randomized to real rTMS and 5 subjects underwent sham rTMS. A two-sample t-test was performed to examine any differences in HIPsub FC alterations between pre-post real rTMS and pre-post sham rTMS. Pre-real-rTMS (or sham-rTMS) maps were subtracted from post-real-rTMS (or sham-rTMS) maps to generate maps of altered FC for each subject. In addition, the interactive effect of group (real group and sham group)×stimulation (before-rTMS and after-rTMS) was explored by a 2×2 repeated-measures ANOVA, with group as the between-subjects factor and stimulation as the within-subject factor for episodic memory, HAMD scores, and HIPsub connectivities.

### Non-parametric statistics

The statistical power of our small sample size was improved by carrying out a re-sampling method comprising stationary bootstrap (10,000 bootstrap samplings) to obtain significant results in demographic data, clinical characteristics, cognitive performance, and FC of HIPsub between baseline assessment and 2 weeks of post-rTMS (sham rTMS) for all statistical analyses (i.e., chi-square test, two-sample t-test, Pearson correlation, and paired-sample t-test). All bootstrap analyses were conducted in SPSS 22.0 software. Supplementary Figure 1 shows the data analysis pipeline conducted in this study.

## Supplementary Material

Supplementary Information
