# Supplementary material for: rTMS modulates precuneus-hippocampal subregion circuit in patients with subjective cognitive decline
Source: Aging (Albany NY). 2020 Nov 30;13(1):1314–31. doi: 10.18632/aging.202313 (PMC7835048; doi:10.18632/aging.202313)
Supplement: Supplementary Information [file aging-13-202313-s001.docx]

# Supplementary Information

## SI methods

### S.1 Data analysis pipeline

### S.2NBH-ADsnp database

### S.3 Neuropsychological assessments for the NBH-ADsnp database

### S.4 Image acquisition for the NBH-ADsnp database

### S.5 fMRI image preprocessing

### S.6 Definition of hippocampal subregions

### S.6 Statistical analysis

## SI results

### S.1 Distinct functional connectivity patterns of HIPsub (HIPe, HIPc, and HIPp)

## SI methods

### S.1 Data analysis pipeline

The study was conducted in two phases: Firstly, the functional connectivity (FC) of HIPsub (HIPe, HIPc, and HIPc) network was evaluated so as to assess altered FC patterns in SCD subjects. Secondly, we analyzed the changes in episodic memory (EM) and the FC of HIPsub in SCD subjects before and after 2 weeks of rTMS treatment in a sham-controlled design. **Supplementary Figure 1** displays the data analysis pipeline conducted in this study.


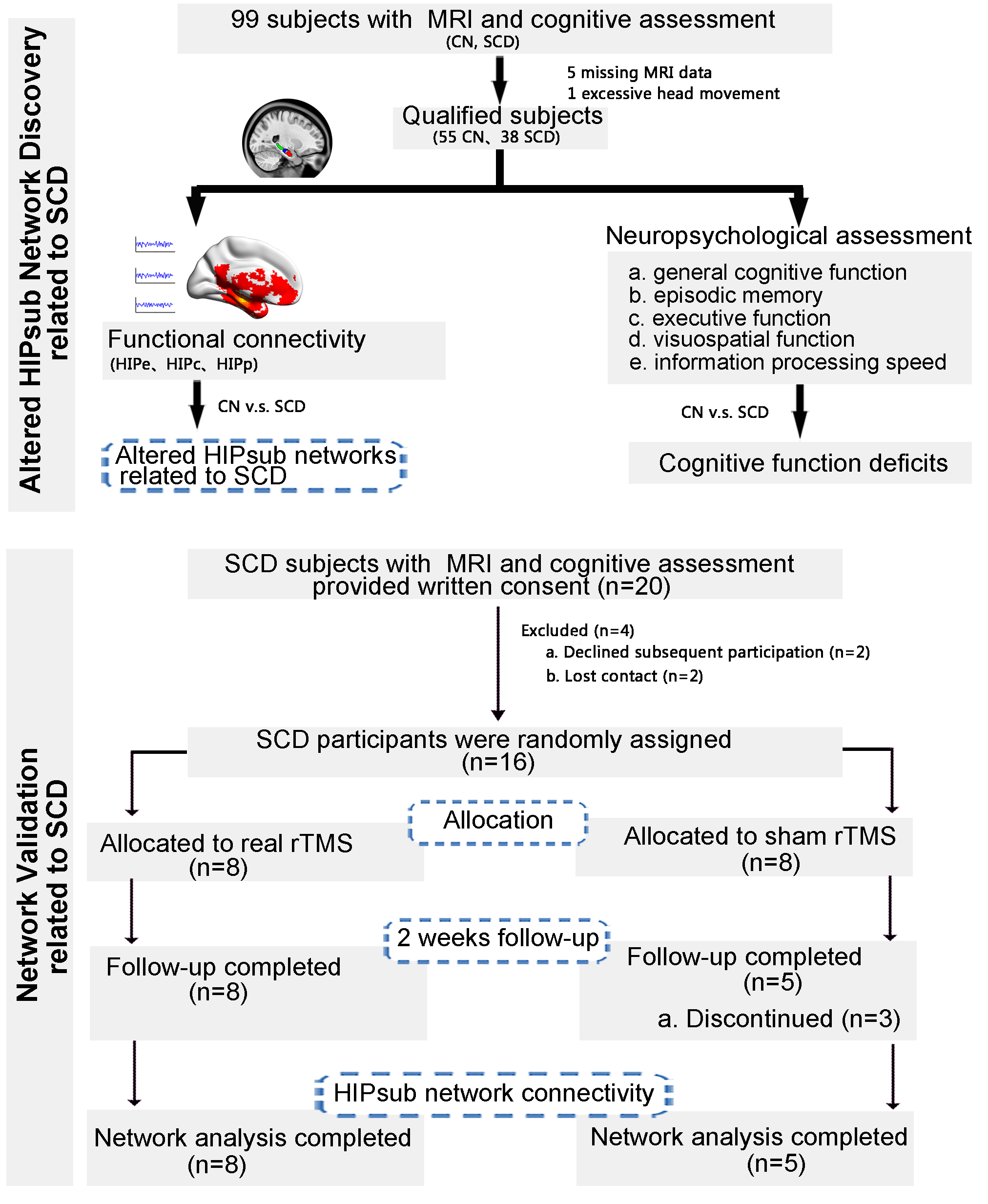


**Supplementary Figure 1.** Schematic of the data analysis pipeline.

**Abbreviations**: CN, healthy controls; SCD, subjective cognitive decline; MRI, magnetic resonance imaging; HIPsub, hippocampal subregion; HIPe, hippocampal emotional region; HIPc, hippocampal cognitive region; HIPp, hippocampal perceptual region.

### S.2NBH-ADsnp database for the NBH-ADsnp database

Data used in this study were obtained from the Nanjing Brain Hospital-Alzheimer’s Disease (AD) Spectrum Neuroimaging Project (NBH-ADsnp) database (in-home website: http://192.168.8.100) (Nanjing, China). NBH-ADsnp was derived from the AD Spectrum Neuroimaging Project that was launched in January 2018 by the Institute of Brain Functional Imaging, the Affiliated Brain Hospital of Nanjing Medical University (Nanjing, China). NBH-ADsnp was initiated by Prof. Jiu Chen (MD, PhD) and Prof. Xiangrong Zhang (MD, PhD), both from the Affiliated Brain Hospital of Nanjing Medical University. Prof. Jiu Chenserved as the principal investigator of NBH-ADsnp. The database was named by Dr. Jiu Chen's research group (discussed by Chen Xue, Guanjie Hu, Wenwen Xu, Wan Liu, Wenzhang Qi, Siyu Wang, Jiani Xu, Shanshan Chen, and finally verified by both Jiu Chen and Xiangrong Zhang).

NBH-ADsnp is an observational study, which includes cross-sectional and longitudinal follow-up components. The goal of NBH-ADsnp was to identify early neuroimaging biomarkers in the preclinical AD spectrum [subjective cognitive decline (SCD), amnestic mild cognitive impairment (aMCI), amnestic mild cognitive impairment (naMCI), and AD] in order to predict disease progression within the spectrum, and to provide imaging-based targets for individualized intervention so as to prevent disease deterioration from preclinical to advanced stages of AD. Initially, several hundreds of older adults, all Han Chinese and right-handed, were recruited from varioushospitals and local communities by word-of-mouth advertisements. The database used a standardized clinical evaluation protocol that includes a medical history interview, neurologic examination, a battery of neurocognitive assessments, and a resting-state MRI scan (T1, T2, 3D T1, DTI, and BOLD) for all participants (healthy controls (CN), SCD, naMCI, aMCI, and AD). All subjects and their study partners completed the informed consent process. The protocols of the study were reviewed and approved by the responsible Human Participants Ethics Committee of the Affiliated Brain Hospital of Nanjing Medical University (No. 2018-KY010-01, No. 2020-KY010-02, and No. ChiCTR1900022287).

**Inclusion and Exclusion criteria**

Inclusion criteria for SCD subjects were based on published SCD criteria, as proposed by the Subjective Cognitive Decline Initiative (SCD-I) [1], which we also described in our previously published article[2]. The inclusion criteria for our database is as follows: (a) self-reported persistent memory decline, which was confirmed by an informant; (b) a Subjective Cognitive Decline Questionnaire (SCD-Q) score > 5 [3-5]; (c) performance within the normal range on MMSE and MoCA (adjusted for age and education); (d) Clinical Dementia Rating (CDR) = 0; (e) subjects aged between 50 and 80 years old;and (f) HAMD scores of less than 7.

Inclusion criteria fornaMCI subjects were as described in prior studies [2, 6]: a) normal overall cognitive function similar to aMCI patients; b) memory function tests scores in the normal range, but with deficits in other cognitive domains, including visual spatial function, executive function, language function, and information processing speed;(c) subjects aged between 50 and 80 years old; and (d) HAMD scores of less than 7.

Inclusion criteria for aMCI subjects were as per the diagnostic criteria as defined by Peterson et al. [7], the revised consensus standards presented by Winblad et al. [8], and the criteria as described in our preceding studies [2, 9, 10] which comprised of the following: (a) a memory complaint, preferably corroborated by an informant or the subject for more than 3 months; (b) objective memory impairment, adjusted for age and educational level; (c) normal general cognitive function of MMSE score equal or above 24; (d) none or minimal impairment in daily living activities; (e) CDR=0.5; (f) subjects aged between 50 and 80 years old; (g) absence of dementia symptoms that were not sufficient to meet the criteria of the National Institute of Neurological and Communicative Disorders and Stroke or the AD and Related Disorders Association criteria for AD; and (h) HAMD scores of less than 7.

Inclusion criteria for CN were as follows: (a) no memory complaints; (b) normal cognitive performance matched for age and education; (c) CDR=0; (d) MMSE ≥ 26; and (e) subjects aged between 50 and 80 years old[2, 11]; and (f) HAMD scores of less than 7.

All participants met the following exclusion criteria, as described in our earlier studies[2, 9, 10]: (a) a past history of stroke (modified HachinskiIschemic Scale Score of > 4), alcoholism, head injury, brain tumors, Parkinson’s disease, epilepsy, encephalitis, major depression (excluded by HAMD), or other neurological or psychiatric illness, as evaluated by clinical assessment and case history; (b) any major medical illness (e.g., cancer, anemia, thyroid dysfunction, syphilis, or HIV); (c) severe visual or hearing loss; (d) inability to complete neuropsychological tests or with a contraindication for MRI; (5) T2-weighted MRI showing major changes in white matter (WM), infarction, or other lesions, as assessed by two experienced radiologists; and (6) no history or current use of psychotropic medications.

### S.3 Neuropsychological assessments for the NBH-ADsnp database

Neuropsychological assessments were performed as described in our previous research papers [2, 9, 11-13]. All subjects had a standardized clinical interview and underwent comprehensive neuropsychological assessments by 3 neuropsychologists (Dr. Xue, Qi, and Liu). The evaluation included the Mini Mental State Examination (MMSE), Montreal Cognitive Assessment (MoCA), Mattis Dementia Rating Scale (MDRS), Auditory Verbal Learning Test - immediate recall (AVLT-IR), Auditory Verbal Learning Test – 5-min delayed recall (AVLT-5-min-DR), Auditory Verbal Learning Test –20-min delayed recall (AVLT-20-min-DR), Logical Memory Test –immediate recall (LMT-IR), Logical Memory Test –20-min delayed recall (LMT-20-min-DR), Rey-Osterrieth Complex Figure Test –20-min delayed recall (ROCFT-20min-DR), Clock Drawing Test (CDT), Rey-Osterrieth Complex Figure Test (ROCFT), Verbal Fluency Test (VFT), Digit Span Test (DST), Digital Symbol Substitution Test (DSST), Trail-Making Tests A and B (TMT-A and B), Stroop Color and Word Test A, B, and C, and Semantic Similarity (Similarity) test. These tests were used to evaluate general cognitive function, episodic memory, information processing speed, executive function, and visuo-spatial function.

### S.4 Image acquisition

The NBH-ADsnp data acquisition process was also depicted in our former articles[2, 9, 10, 14]. All MRI data were acquired using a 3.0 Tesla Verio Siemens scanner with an 8-channel head-coil at the Affiliated Brain Hospital of Nanjing Medical University (Nanjing, China). Resting-state functional images were collected while participants were instructed to rest with their eyes open, not to fall asleep, and not to think of anything in particular. The gradient-echo echo-planar imaging (GRE-EPI) sequence included 240 volumes. The parameters were as follows: repetition time (TR) = 2000 ms, echo time (TE) = 30 ms，number of slices = 36, thickness = 4.0 mm, gap = 0 mm, matrix = 64×64, flip angle (FA) = 90°, field of view (FOV) = 220 mm×220 mm, acquisition bandwidth = 100 kHz, voxel size = 3.4×3.4×4 mm^3^. The imaging process took approximately 8 minutes.

High-resolution T1-weighted images were obtained by a 3D magnetization-prepared rapid gradient-echo (MPRAGE) sequence. The parameters were as follows: TR = 1900 ms, TE = 2.48 ms, inversion time (TI) = 900 ms, number of slices = 176, thickness = 1.0 mm, gap = 0.5 mm, matrix = 256×256, FA = 9°, FOV = 256 mm×256 mm, voxel size = 1×1×1 mm^3^. The imaging processlasted for approximately 4.26 minutes.

Moreover, routine axial T2-weighted images were acquired to rule out subjects with major changes in white matter (WM), cerebral infarction or other lesions using flair sequence as follows: TR = 8400 ms, TE = 94 ms, FA= 150°, acquisition matrix = 256×256, FOV = 230×230 mm, thickness = 5.0 mm, gap = 0 mm, and number of slices = 20. The imaging processtook approximately 2.50 minutes to complete.

### S.5 fMRI image preprocessing

The image processing procedures were performed as previously described by Yan et al.[15]. All fMRI data were preprocessed using MATLAB 2015b (http://www.mathworks.com/products/matlab/) and DPABI image processing software[16]. The first ten volumes were discarded in order to reduce the instability of MRI signal. Corrections were performed for the intra-volume acquisition time differences among slices and inter-volume motion effects during the scan (slice timing correction and head motion correction) [17, 18]. Participants with excessive head motion (cumulative translation or rotation > 3.0 mm or 3.0°) were excluded from further analyses. Individual functional and structural images were then co-registered. We used the Diffeomorphic Anatomical Registration Through Exponentiated Lie Algebra (DARTEL) algorithm to normalize and segment the structural images into GM, WM and cerebrospinal fluid (CSF) partitions[19]. Next, weused a Friston 24-parameter model (i.e., 6 head motion parameters, 6 head motion parameters one time point before, and the 12 corresponding squared items) to regress out head motion effects from the realigned data [20]. The WM, CSF, global signals and linear trend wereregressed as nuisance covariates [21]. After realignment, slice timing correction, and co-registration, framewise displacement (FD) was calculated for all resting state volumes [17]. All volumes with a FD greater than 0.2 mm were regressed out as nuisance covariates [21]. Any scan with 50% of volumes removed was additionally discarded [21]. After nuisance covariate regression, functional images were normalized by DARTEL into MNI space (resampling voxel size, 3 × 3 × 3 mm³) and then spatially smoothed by a Gaussian kernel of 6 mm^3^ full-width at half maximum (FWHM) to reduce spatial noise[10]. Temporal band-pass filtering (0.01–0.1 Hz) was applied to reduce the effect of low-frequency drifts and high-frequency physiological noise[15, 22]. Voxels within a group GM mask, created by DARTEL, were used for further analyses.

### S.6 Definition of hippocampal subregions

The definition of HIPsub employed throughout our study was originally designated by Robinson et al. [23] and Bai et al.[24], who used coactivation-based parcellation to reveal a subspecialization in the hippocampus by a data-driven method. Therefore, we selected only the left HIPsub as regions of interest (ROI) (Supplementary Figure 2A) based on these past studies[24]. In the current article, the left hippocampus was defined as three subregions locatedon the anterior part involved in emotional processes (HIPe), middle part involved in cognitive processes (HIPc), and posterior part involved in perceptual function (HIPp). It's worth noting that we removed the overlapping areas between adjacent clusters (Supplementary Figure 2B) as presented by Bai et al. [24], which could have affected the subsequent analysis. Non-overlapping clusters were then selected as ROIs for further analysis (Supplementary Figure 2C).


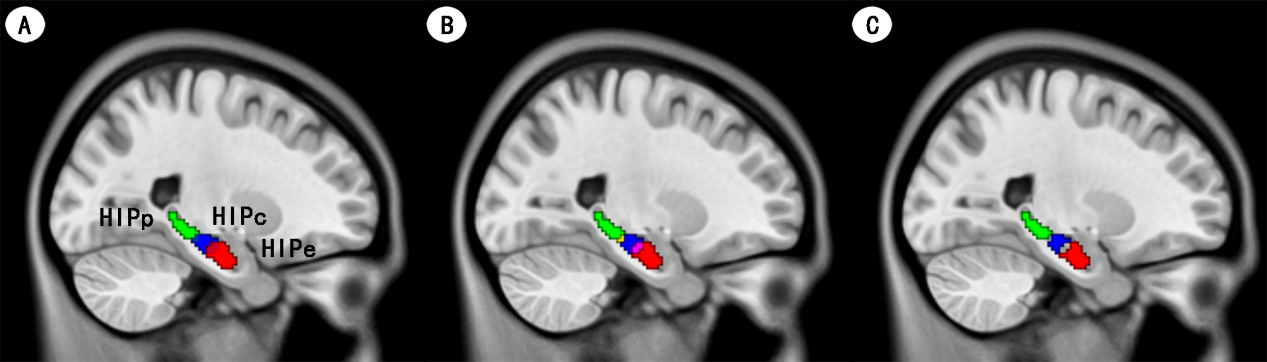


**Supplementary Figure 2**. Schematic diagram of hippocampal subregions (sagittal views) in the left hemisphere. (A) The selected hippocampal subregions were based on recent studies published by Robinson et al. [23] and Bai et al.[24], who used coactivation-based parcellation to reveal a subspecialization in the hippocampus by a data-driven method. Hippocampal subregions included HIPe (blue), HIPc (red), and HIPp (green). (B) The magenta area and yellow area indicated overlapping areas between HIPe and HIPc, and between HIPc and HIPp, respectively. (C) HIPe, HIPc, and HIPpwere considered for further analysis. Abbreviations: HIPe, hippocampal emotional region; HIPc, hippocampal cognitive region; HIPp, hippocampal perceptual region.

### S.7 Statistical analysis

The present study combined neuropsychological tests into 4 cognitive domains and transformed the raw scores into 4 composite Z scores [10, 13] in order to increase statistical power by reducing random variability. First, for each neuropsychological test, the individual raw scores were transformed to Z scores, according to the mean and standard deviation for all subjects. Notably, for tests measured by timing, including TMT-A, TMT-B, Stroop A, Stroop B, and Stroop C, the raw scores were defined as the reciprocal of the time required for the test. Then, each cognitive domain’s composite Z score was determined by averaging the Z scores related to the tests. We divided these tests into 4 cognitive domains: episodic memory (3 tests, including AVLT-20-min DR, LMT-20-min DR, and CFT-20-min DR), information processing speed (4 tests, comprising DSST, TMT-A, Stroop A, and Stroop B), visuospatial function (2 tests, including CFT and CDT), and executive function (5 tests, consisting of VFT, DST-backward, TMT-B, Stroop C, and Similarity).

## SI results

### S.1 Distinct functional connectivity patterns of HIPsub (HIPe, HIPc, and HIPp)

***Supplementary Figure 3*** shows distinct functional connectivity patterns of HIPe, HIPc, and HIPp.


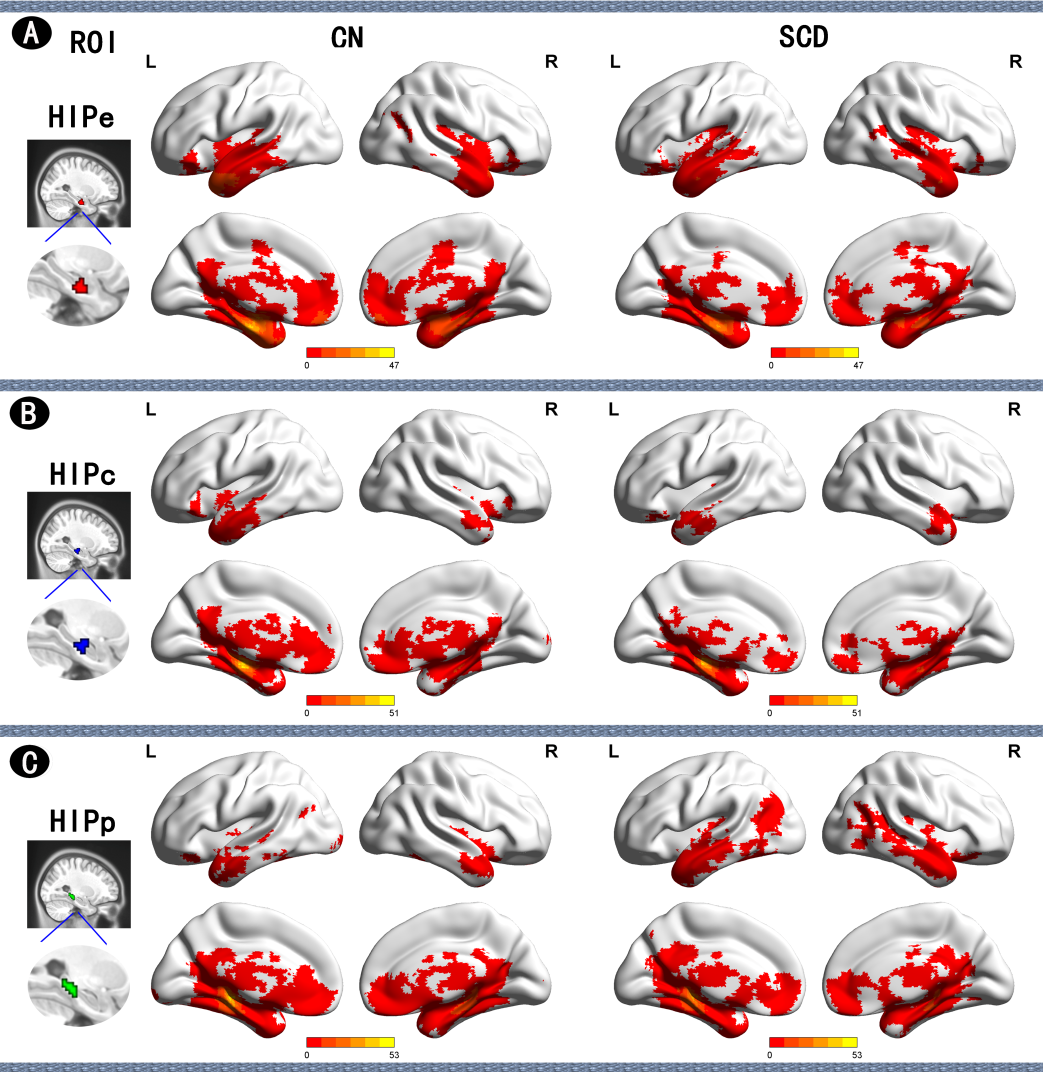


**Supplementary Figure 3.** Seed-based intrinsic connectivity maps derived from hippocampal-subregion seeds (HIPe, HIPc, and HIPp) in CN and SCD subjects. Results from one-sample T-test are displayed using TFCE-FWE-corrected p < 0.001 and cluster extent k > 100 voxels (2700 mm^3^).A, B, and Cindicate intrinsic connectivity maps derived from HIPe, HIPc, and HIPp, respectively.

**Abbreviations**: CN, healthy controls; SCD, subjective cognitive decline; HIPe, hippocampal emotional region; HIPc, hippocampal cognitive region; HIPp, hippocampal perceptual region; L, left hemisphere; R, right hemisphere, ROI, region of interest. TFCE, threshold-free cluster enhancement; FWE, family wise error.

## S.2 Changes of depression scores pre- v.s. post-rTMS (or sham rTMS)





**Supplementary Figure 4.** Changes of depression scores before and after real-rTMS or sham-rTMS.

**Abbreviations**: SCD, subjective cognitive decline; HAMD, Hamilton Depression Scale; rTMS, repetitive transcranial magnetic stimulation.

## Supplementary tables

### Supplementary Table 1 (related to Table 1). Raw scores and corresponding Z scores of individual neuropsychological tests for all subjects.

| Items |  | CN |  | SCD |
| --- | --- | --- | --- | --- |
|  |  | n=55 |  | n=38 |
| **MDRS** |  |  |  |  |
| Attention | raw score | 36.75(0.52) |  | 36.53(0.73) |
|  | *Z* score | 0.29(0.38) |  | 0.13(0.54) |
| Initiation/Preservation | raw score | 36.60(1.24) |  | 36.66(1.58) |
|  | *Z* score | 0.18(0.57) |  | 0.21(0.73) |
| Conceptual | raw score | 38.42(1.01) |  | 38.37(1.05) |
|  | *Z* score | 0.22(0.53) |  | 0.19(0.55) |
| Construct | raw score | 5.98(0.13) |  | 5.87(0.41) |
|  | *Z* score | 0.20(0.31) |  | -0.06(0.95) |
| Memory | raw score | 23.71(1.29) |  | 22.92(1.75) |
|  | *Z* score | 0.46(0.67) |  | 0.05(0.91) |
| Total | raw score | 141.46(2.33) |  | 140.37(3.05) |
|  | *Z* score | 0.42(0.46) |  | 0.21(0.60) |
| **Episodic memory** |  |  |  |  |
| AVLT-IM | raw score | 19.15(4.36) |  | 18.66(4.22) |
|  | *Z* score | 0.35(0.94) |  | 0.25(0.91) |
| AVLT-5-min-DR | raw score | 6.35(2.20) |  | 6.26(1.90) |
|  | *Z* score | 0.34(0.93) |  | 0.31(0.80) |
| AVLT-20-min-DR | raw score | 6.30(1.94) |  | 6.32(2.12) |
|  | *Z* score | 0.40(0.73) |  | 0.41(0.80) |
| LMT-IR | raw score | 6.29(3.25) |  | 5.68(3.31) |
|  | *Z* score | 0.30(1.00) |  | 0.11(1.02) |
| LMT-20-min-DR | raw score | 4.95(3.07) |  | 4.82(2.83) |
|  | *Z* score | 0.26(1.06) |  | 0.22(0.98) |
| ROCFT-20-min-DR | raw score | 16.51(5.49) |  | 18.04(6.15) |
|  | *Z* score | 0.15(0.83) |  | 0.38(0.93) |
| **Visuospatial function** |  |  |  |  |
| ROCFT | raw score | 34.78(2.11) |  | 35.00(1.43) |
|  | *Z* score | 0.20(0.78) |  | 0.28(0.53) |
| CDT | raw score | 9.36(1.21) |  | 9.47(0.95) |
|  | *Z* score | 0.14(1.03) |  | 0.24(0.81) |
| **Information processing speed** |  |  |  |  |
| DSST | raw score | 44.16(10.23) |  | 42.42(11.40) |
|  | *Z* score | 0.39(0.90) |  | 0.23(1.01) |
| TMT-A | raw score | 51.62(12.80) |  | 53.68(13.98) |
|  | *Z* score | 0.31(1.09) |  | 0.15(0.84) |
| Stoop-A | raw score | 25.06(4.69) |  | 25.74(5.12) |
|  | *Z* score | 0.22(0.91) |  | 0.12(1.12) |
| Stoop-B | raw score | 41.69(9.85) |  | 41.40(11.22) |
|  | *Z* score | 0.15(0.93) |  | 0.21(0.96) |
| **Executive function** |  |  |  |  |
| VFT | raw score | 26.27(6.71) |  | 26.26(6.59) |
|  | *Z* score | 0.19(0.93) |  | 0.19(0.91) |
| DST | raw score | 12.86(1.70) |  | 13.00(1.71) |
|  | *Z* score | 0.28(0.90) |  | 0.35(0.91) |
| TMT-B | raw score | 122.56(31.45) |  | 135.32 (42.43) |
|  | *Z* score | 0.39(0.86) |  | 0.18(1.05) |
| Stoop-C | raw score | 77.62(20.25) |  | 74.68(21.64) |
|  | *Z* score | 0.14(0.94) |  | 0.33(1.05) |
| Similarity | raw score | 19.06(3.52) |  | 19.50(3.21) |
|  | *Z* score | 0.34(0.77) |  | 0.44(0.70) |

Data are presented as the mean (standard deviation, SD). **Abbreviations**: CN, healthy controls; SCD, subjective cognitive decline; MDRS, Mattis Dementia Rating Scale; AVLT-IR, Auditory Verbal Learning Test – immediate recall; AVLT-5-min-DR, Auditory Verbal Learning Test – 5-minute delayed recall; AVLT-20-min-DR, Auditory Verbal Learning Test – 20-minute delayed recall; LMT-IR, Logical Memory Test – immediate recall; LMT-20-min-DR, Logical Memory Test – 20-minute delayed recall; ROCFT-20min-DR, Rey-Osterrieth Complex Figure Test – 20-minute delayed recall; CDT, Clock Drawing Test; ROCFT, Rey-Osterrieth Complex Figure Test; DSST, Digital Symbol Substitution Test; TMT-A, Trail Making Test-A; Stroop, Stroop Color and Word Test; VFT, Verbal Fluency Test; DST, Digit Span Test; TMT-B, Trail Making Test-B; Similarity, Semantic Similarity Test. In Supplementary Table 1, no items had significant group differences.

### Supplementary Table 2. Demographic characteristics, clinical measures, and episodic memory of SCD before real and sham rTMS treatment.

| **Items** | **real rTMS SCD** |  | **sham rTMS SCD** |
| --- | --- | --- | --- |
|  | **n=8** |  | **n=5** |
| Age (years) | 70.00(6.37) |  | 72.20(5.12) |
| Gender (male/female) | 4/4 |  | 2/3 |
| Education level (years) | 12.13(3.56) |  | 13.20(2.78) |
| MMSE | 28.50(1.60) |  | 28.00(1.87) |
| MoCA | 25.25(1.83) |  | 24.80(1.48) |
| MDRS | 141.00(3.16) |  | 141.80(1.30) |
| SCD-Q | 6.69(0.92) |  | 6.80(1.20) |
| **Episodic memory tests** |  |  |  |
| AVLT-IR | 17.25(4.43) |  | 15.00(2.83) |
| AVLT-5min-DR | 6.00(2.39) |  | 4.60(1.52) |
| AVLT-20min-DR | 6.38(2.93) |  | 4.20(1.30) |
| **AVLT-total** | 29.88(9.16) |  | 24.20(4.82) |
| LM-IR | 5.00(2.62) |  | 3.80(1.09) |
| LM-20-min-DR | 3.63(3.34) |  | 2.80(1.92) |
| ROCFT-20-min-DR | 18.63(6.39) |  | 17.40(4.67) |
| Total scores | 56.88(14.21) |  | 47.80(7.43) |

Data are presented as the mean (standard deviation, SD). **Abbreviations**: SCD, subjective cognitive decline; MMSE, Mini Mental State Examination; MoCA, Montreal Cognitive Assessment; MDRS, Mattis Dementia Rating Scale; SCD-Q, Subjective Cognitive Decline-Questionnaire; AVLT-IR, Auditory Verbal Learning Test – immediate recall; AVLT-5min-DR, Auditory Verbal Learning Test – 5-minute delayed recall; AVLT-20min-DR, Auditory Verbal Learning Test – 20-minute delayed recall; LMT-IR, Logical Memory Test – immediate recall; LMT-20-min-DR, Logical Memory Test – 20-minute delayed recall; ROCFT-20min-DR, Rey-Osterrieth Complex Figure Test – 20-minute delayed recall.

### Supplementary Table 3 (related to Figure 4). Functional connectivity comparisons of the HIPsub seeds between SCD and CN subjects.

| **Brain regions** | **L/R** | |  | **BA** | **MNI** | | | ***F*/*T* values** | **Cluster size (mm^3^)** |
| --- | --- | --- | --- | --- | --- | --- | --- | --- | --- |
|  |  |  |  |  | **x** | **y** | **z** |  |  |
| **HIPefuncational connectivity** | | | | | | | | | |
| **CN v.s. SCD** |  | |  |  |  | | |  |  |
| Cerebellum Posterior Lobe | R | |  | - | 6 -51 -45 | | | 3.2927 | 2187 |
| Fusiform Gyrus | L | |  | 18 | -36 -51 -24 | | | -3.2698 | 648 |
| Insula | L | |  | 13 | -39 -18 18 | | | -4.0272 | 513 |
| Parahippocampa Gyrus | L | |  | 34 | -21 -9 -15 | | | -3.7454 | 2214 |
| **HIPcfuncational connectivity** | | | | | | | | | |
| **CN v.s. SCD** |  |  | |  |  | | |  |  |
| Parahippocampa Gyrus | L |  | | 34 | -30 -15 -24 | | | -3.4989 | 1350 |
| Inferior Frontal Gyrus, orbital part | R |  | | 47 | 45 27 -6 | | | 3.714 | 1323 |
| **HIPpfuncational connectivity** | | | | | | | | | |
| **CN v.s. SCD** |  |  | |  |  | | |  |  |
| Middle Temporal Gyrus | R |  | | 21 | 48 -81 15 | | | -3.4392 | 8937 |
| Middle Temporal Gyrus | L |  | | 21 | -45 -60 -3 | | | -3.8487 | 9234 |
| Cingulate Gyrus/Precuneus | L |  | | 31 | -12 -45 36 | | | -2.4823 | 405 |
| Insula | L |  | | 13 | -30 -33 0 | | | -3.5957 | 6372 |
| Medial Frontal Gyrus | L |  | | 11 | -6 27 -21 | | | 2.778 | 1350 |

**Abbreviations**: CN, healthy controls; SCD, subjective cognitive decline; HIPe, hippocampal emotional region; HIPc, hippocampal cognitive region; HIPp, hippocampal perceptual region; MNI, montreal neurological institute; L, left hemisphere; R, right hemisphere.

**References**

1. Jessen F, Amariglio RE, van Boxtel M, Breteler M, Ceccaldi M, Chételat G, Dubois B, Dufouil C, Ellis KA, van der Flier WM, Glodzik L, van Harten AC, de Leon MJ, et al. A conceptual framework for research on subjective cognitive decline in preclinical Alzheimer's disease. Alzheimers Dement. 2014; 10:844–52.

<https://doi.org/10.1016/j.jalz.2014.01.001> PMID:[24798886](https://pubmed.ncbi.nlm.nih.gov/24798886)

2. Xue C, Yuan B, Yue Y, Xu J, Wang S, Wu M, Ji N, Zhou X, Zhao Y, Rao J, Yang W, Xiao C, Chen J. Distinct disruptive patterns of default mode subnetwork connectivity across the spectrum of preclinical Alzheimer’s disease. Front Aging Neurosci. 2019; 11:307.

<https://doi.org/10.3389/fnagi.2019.00307> PMID:[31798440](https://pubmed.ncbi.nlm.nih.gov/31798440)

3. Yan T, Wang W, Yang L, Chen K, Chen R, Han Y. Rich club disturbances of the human connectome from subjective cognitive decline to Alzheimer’s disease. Theranostics. 2018; 8:3237–55.

<https://doi.org/10.7150/thno.23772> PMID:[29930726](https://pubmed.ncbi.nlm.nih.gov/29930726)

4. Cedres N, Machado A, Molina Y, Diaz-Galvan P, Hernández-Cabrera JA, Barroso J, Westman E, Ferreira D. Subjective cognitive decline below and above the age of 60: a multivariate study on neuroimaging, cognitive, clinical, and demographic measures. J Alzheimers Dis. 2019; 68:295–309.

<https://doi.org/10.3233/JAD-180720> PMID:[30741680](https://pubmed.ncbi.nlm.nih.gov/30741680)

5. Hao L, Wang X, Zhang L, Xing Y, Guo Q, Hu X, Mu B, Chen Y, Chen G, Cao J, Zhi X, Liu J, Li X, et al. Prevalence, risk factors, and complaints screening tool exploration of subjective cognitive decline in a large cohort of the Chinese population. J Alzheimers Dis. 2017; 60:371–88.

<https://doi.org/10.3233/JAD-170347> PMID:[28869471](https://pubmed.ncbi.nlm.nih.gov/28869471)

6. Dunn CJ, Duffy SL, Hickie IB, Lagopoulos J, Lewis SJ, Naismith SL, Shine JM. Deficits in episodic memory retrieval reveal impaired default mode network connectivity in amnestic mild cognitive impairment. Neuroimage Clin. 2014; 4:473–80.

<https://doi.org/10.1016/j.nicl.2014.02.010> PMID:[24634833](https://pubmed.ncbi.nlm.nih.gov/24634833)

7. Petersen RC, Smith GE, Waring SC, Ivnik RJ, Tangalos EG, Kokmen E. Mild cognitive impairment: clinical characterization and outcome. Arch Neurol. 1999; 56:303–08.

<https://doi.org/10.1001/archneur.56.3.303> PMID:[10190820](https://pubmed.ncbi.nlm.nih.gov/10190820)

8. Winblad B, Palmer K, Kivipelto M, Jelic V, Fratiglioni L, Wahlund LO, Nordberg A, Bäckman L, Albert M, Almkvist O, Arai H, Basun H, Blennow K, et al. Mild cognitive impairment—beyond controversies, towards a consensus: report of the international working group on mild cognitive impairment. J Intern Med. 2004; 256:240–46.

<https://doi.org/10.1111/j.1365-2796.2004.01380.x> PMID:[15324367](https://pubmed.ncbi.nlm.nih.gov/15324367)

9. Chen J, Chen G, Shu H, Chen G, Ward BD, Wang Z, Liu D, Antuono PG, Li SJ, Zhang Z, and Alzheimer’s Disease Neuroimaging Initiative. Predicting progression from mild cognitive impairment to Alzheimer’s disease on an individual subject basis by applying the CARE index across different independent cohorts. Aging (Albany NY). 2019; 11:2185–201.

<https://doi.org/10.18632/aging.101883> PMID:[31078129](https://pubmed.ncbi.nlm.nih.gov/31078129)

10. Chen J, Shu H, Wang Z, Zhan Y, Liu D, Liao W, Xu L, Liu Y, Zhang Z. Convergent and divergent intranetwork and internetwork connectivity patterns in patients with remitted late-life depression and amnestic mild cognitive impairment. Cortex. 2016; 83:194–211.

<https://doi.org/10.1016/j.cortex.2016.08.001> PMID:[27570050](https://pubmed.ncbi.nlm.nih.gov/27570050)

11. Chen J, Shu H, Wang Z, Zhan Y, Liu D, Liu Y, Zhang Z. Intrinsic connectivity identifies the sensory-motor network as a main cross-network between remitted late-life depression- and amnestic mild cognitive impairment-targeted networks. Brain Imaging Behav. 2020; 14:1130–42.

<https://doi.org/10.1007/s11682-019-00098-4> PMID:[31011952](https://pubmed.ncbi.nlm.nih.gov/31011952)

12. Chen J, Shu H, Wang Z, Liu D, Shi Y, Zhang X, Zhang Z. The interaction of APOE genotype by age in amnestic mild cognitive impairment: a voxel-based morphometric study. J Alzheimers Dis. 2015; 43:657–68.

<https://doi.org/10.3233/JAD-141677> PMID:[25114090](https://pubmed.ncbi.nlm.nih.gov/25114090)

13. Chen J, Duan X, Shu H, Wang Z, Long Z, Liu D, Liao W, Shi Y, Chen H, Zhang Z. Differential contributions of subregions of medial temporal lobe to memory system in amnestic mild cognitive impairment: insights from fMRI study. Sci Rep. 2016; 6:26148.

<https://doi.org/10.1038/srep26148> PMID:[27184985](https://pubmed.ncbi.nlm.nih.gov/27184985)

14. Chen J, Yan Y, Gu L, Gao L, Zhang Z. Electrophysiological processes on motor imagery mediate the association between increased gray matter volume and cognition in amnestic mild cognitive impairment. Brain Topogr. 2020; 33:255–66.

<https://doi.org/10.1007/s10548-019-00742-8> PMID:[31691911](https://pubmed.ncbi.nlm.nih.gov/31691911)

15. Yan CG, Craddock RC, Zuo XN, Zang YF, Milham MP. Standardizing the intrinsic brain: towards robust measurement of inter-individual variation in 1000 functional connectomes. Neuroimage. 2013; 80:246–62.

<https://doi.org/10.1016/j.neuroimage.2013.04.081> PMID:[23631983](https://pubmed.ncbi.nlm.nih.gov/23631983)

16. Yan CG, Wang XD, Zuo XN, Zang YF. DPABI: data processing & analysis for (resting-state) brain imaging. Neuroinformatics. 2016; 14:339–51.

<https://doi.org/10.1007/s12021-016-9299-4> PMID:[27075850](https://pubmed.ncbi.nlm.nih.gov/27075850)

17. Power JD, Barnes KA, Snyder AZ, Schlaggar BL, Petersen SE. Spurious but systematic correlations in functional connectivity MRI networks arise from subject motion. Neuroimage. 2012; 59:2142–54.

<https://doi.org/10.1016/j.neuroimage.2011.10.018> PMID:[22019881](https://pubmed.ncbi.nlm.nih.gov/22019881)

18. Van Dijk KR, Sabuncu MR, Buckner RL. The influence of head motion on intrinsic functional connectivity MRI. Neuroimage. 2012; 59:431–38.

<https://doi.org/10.1016/j.neuroimage.2011.07.044> PMID:[21810475](https://pubmed.ncbi.nlm.nih.gov/21810475)

19. Ashburner J, Friston KJ. Computing average shaped tissue probability templates. Neuroimage. 2009; 45:333–41.

<https://doi.org/10.1016/j.neuroimage.2008.12.008> PMID:[19146961](https://pubmed.ncbi.nlm.nih.gov/19146961)

20. Friston KJ, Williams S, Howard R, Frackowiak RS, Turner R. Movement-related effects in fMRI time-series. Magn Reson Med. 1996; 35:346–55.

<https://doi.org/10.1002/mrm.1910350312> PMID:[8699946](https://pubmed.ncbi.nlm.nih.gov/8699946)

21. Brady RO Jr, Gonsalvez I, Lee I, Öngür D, Seidman LJ, Schmahmann JD, Eack SM, Keshavan MS, Pascual-Leone A, Halko MA. Cerebellar-prefrontal network connectivity and negative symptoms in schizophrenia. Am J Psychiatry. 2019; 176:512–20.

<https://doi.org/10.1176/appi.ajp.2018.18040429> PMID:[30696271](https://pubmed.ncbi.nlm.nih.gov/30696271)

22. Zhou C, Yu M, Tang X, Wang X, Zhang X, Zhang X, Chen J. Convergent and divergent altered patterns of default mode network in deficit and non-deficit schizophrenia. Prog Neuropsychopharmacol Biol Psychiatry. 2019; 89:427–34.

<https://doi.org/10.1016/j.pnpbp.2018.10.012> PMID:[30367960](https://pubmed.ncbi.nlm.nih.gov/30367960)

23. Robinson JL, Barron DS, Kirby LA, Bottenhorn KL, Hill AC, Murphy JE, Katz JS, Salibi N, Eickhoff SB, Fox PT. Neurofunctional topography of the human hippocampus. Hum Brain Mapp. 2015; 36:5018–37.

<https://doi.org/10.1002/hbm.22987> PMID:[26350954](https://pubmed.ncbi.nlm.nih.gov/26350954)

24. Bai T, Wei Q, Xie W, Wang A, Wang J, Ji GJ, Wang K, Tian Y. Hippocampal-subregion functional alterations associated with antidepressant effects and cognitive impairments of electroconvulsive therapy. Psychol Med. 2019; 49:1357–64.

<https://doi.org/10.1017/S0033291718002684> PMID:[30229715](https://pubmed.ncbi.nlm.nih.gov/30229715)

## Supplementary table legend

### Supplementary Table 1 (related to Table 1). Raw scores and corresponding Z scores of individual neuropsychological tests for all subjects.

### Supplementary Table 2. Demographic characteristics, clinical measures, and episodic memory of SCD before real and sham rTMS treatment.

### Supplementary Table 3 (related to Figure 4). Functional connectivity comparisons of of the HIPsub seeds between SCD and CN subjects.

## Supplementary figure legend

### Supplementary Figure 1. Schematic of the data analysis pipeline.

### Supplementary Figure 2. Schematic diagram of hippocampal subregionsin the left hemisphere

### Supplementary Figure 3. (related to Figure1-3).Seed-based intrinsic connectivity maps derived from hippocampal-subregion seeds (HIPe, HIPc, aFdHIPp) in CN and SCD subjects.

**Supplementary Figure 4.** Changes of depression scores before and after real-rTMS or sham-rTMS.
